# Short Stature: Think About the Pituitary Stalk Interruption Syndrome

**DOI:** 10.7759/cureus.35700

**Published:** 2023-03-02

**Authors:** El Mehdi Mniai, Abderrahim Bourial, Siham Salam, Mohamed Mahi, Amal Rami

**Affiliations:** 1 Radiology, Cheikh Khalifa International University Hospital, Mohammed VI University of Health Sciences, Casablanca, MAR; 2 Otolaryngology, Cheikh Khalifa International University Hospital, Mohammed VI University of Health Sciences, Casablanca, MAR; 3 Pediatric Radiology, Abderrahim Harouchi Mother-Child Hospital, Casablanca, MAR; 4 Radiology, Hassan II University Ain Chock, Casablanca, MAR

**Keywords:** short stature, pituitary stalk interruption syndrome, pituitary gland abnormalities, pituitary gland, diagnostic imaging, pituitary diseases, growth hormone deficiency, magnetic resonance imaging, hypopituitarism

## Abstract

Pituitary stalk interruption syndrome (PSIS) is an uncommon congenital defect of the pituitary gland. It is considered one of the rare endocrinal causes of abnormally short stature. Herein, we present a case of a four-year-old girl who consulted for short stature and delayed growth. The patient's history did not include any past medical or surgical pathology. Birth history revealed a full-term delivery with a breech presentation. Clinically, the patient had a small stature, beneath the third percentile. Magnetic resonance imaging findings, through a typical triad, were consistent with PSIS. We describe through this report, what we believe is a rare typical case of PSIS. This case was discovered in a young patient with pituitary dwarfism. We hope that the concise and synthesized structure of this case report will help physicians acquire the necessary reflexes to notice and diagnose the already underdiagnosed PSIS.

## Introduction

The adult pituitary gland is generally composed of two lobes: anterior and posterior. The anterior lobe (adenohypophysis) is derived from the oral ectoderm, and the posterior lobe (neurohypophysis) is derived from the neural ectoderm [1]. Pituitary stalk interruption syndrome (PSIS) is an uncommon congenital defect of the pituitary gland. It is considered one of the rare endocrinal causes of abnormally short stature [2]. PSIS is a heterogeneous entity regarding its clinical and hormonal presentation. The main consequence of PSIS is hypopituitarism. The latter is defined by one or multiple deficiencies in pituitary hormones. The diagnosis of PSIS is based on magnetic resonance imaging (MRI) findings [3]. It is characterized by a specific MRI triad, which, if recognized, allows early diagnosis and thus appropriate treatment. We describe through this report, what we believe is a rare typical case of PSIS.

## Case presentation

A four-year-old Moroccan girl consulted for short stature and delayed growth. Her parents noticed that their child was not growing like other children of her age and that she was the smallest of her classmates. The patient's history did not include any past medical or surgical pathology. Birth history revealed a full-term delivery with a breech presentation. On physical examination, the patient did not present any facial abnormality. She was fully oriented to time and space. Respiratory auscultation and heart sounds were normal and so was the abdominal examination. No psychomotor retardation was noted. The patient’s weight was 10.4 kg (below the third percentile), with an estimated optimal weight of 16 kg. In addition, the patient had a short stature with a height of 86 cm (below the third percentile). The optimal height for age and sex was estimated at 101 cm. The parents' heights were, respectively, 165 cm for the mother and 172 cm for the father. The mid-parental height (MPH) was calculated to be 162 cm, which excluded a familial short stature.

**Figure 1:**
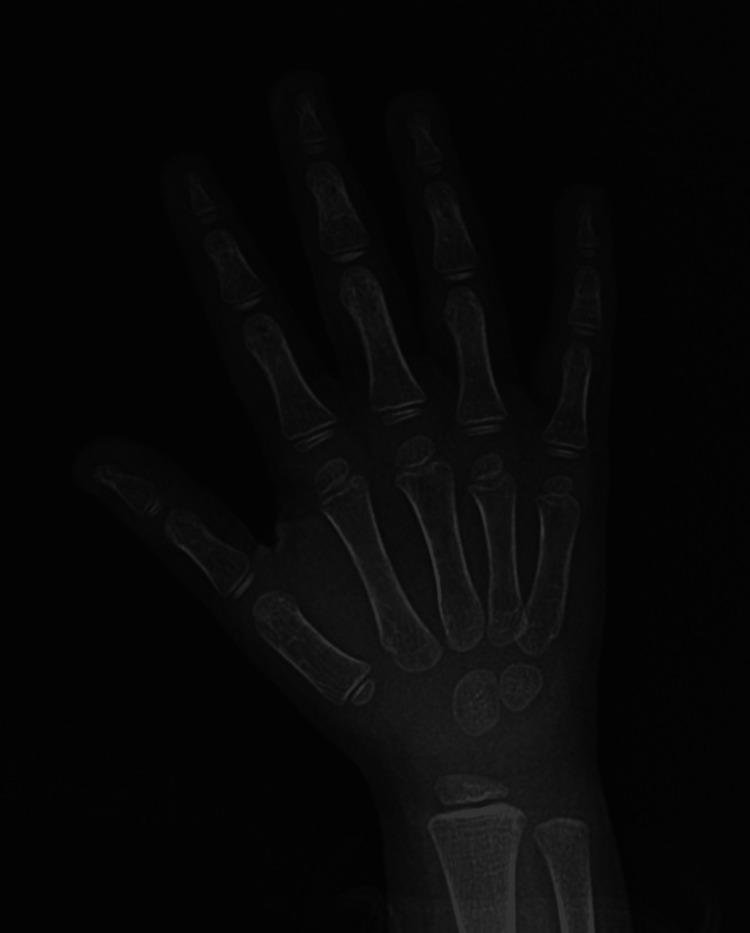
Estimation of bone age of two years according to the Greulich and Pyle method

On laboratory assessment, serum insulin-like growth factor 1 (IGF-1) was assessed at 17.7 ng/mL, lower than the normal level for the age (30-236 ng/mL), whereas all other anterior pituitary hormones were within the normal range. The growth hormone stimulation test revealed growth hormone deficiency. In front of these suggestive clinical and biological features, a central cause of the delayed growth was evoked. According to the Greulich and Pyle method, her bone age was estimated as two years (two years less than her chronological age) (Figure 1).

**Figure 2:**
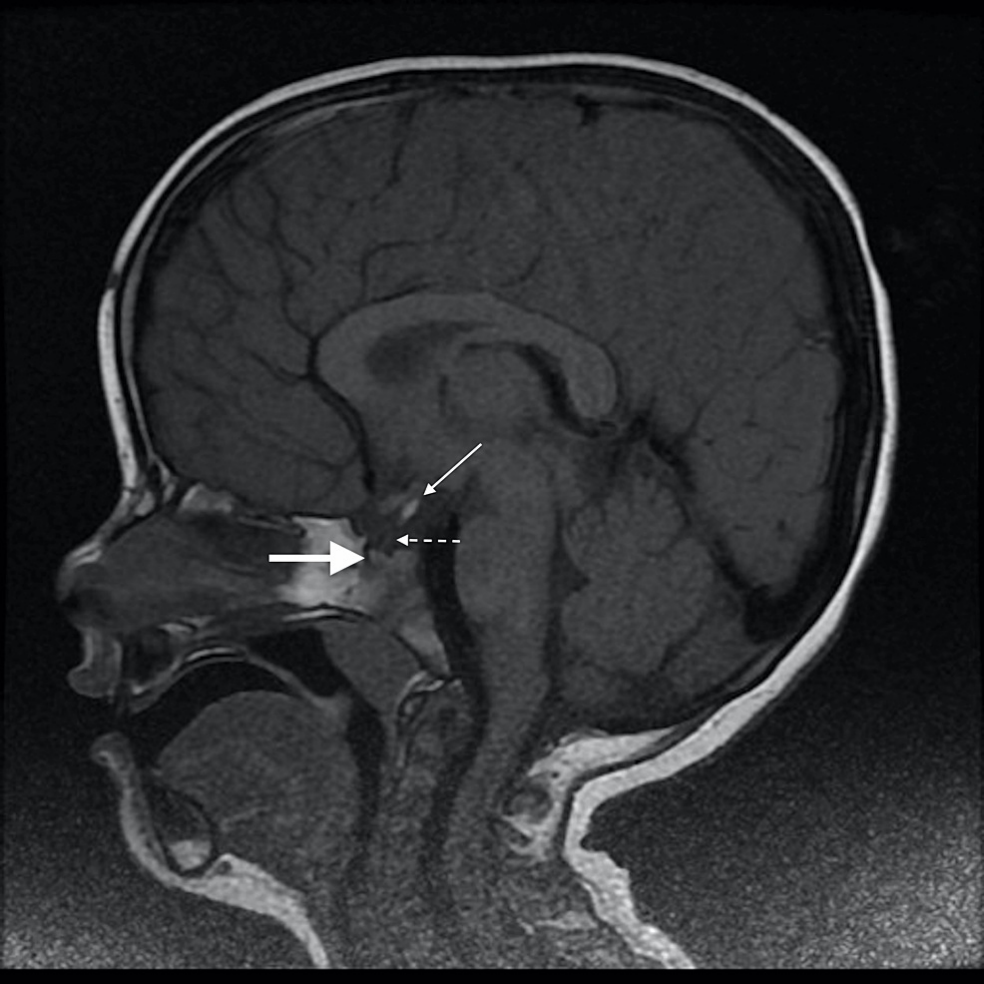
Mid-sagittal T1-weighted MRI section revealing an undersized sella turcica with a hypoplastic adenohypophysis (thick white arrow), a hypoplastic pituitary stalk (dashed line arrow), and an ectopic posterior pituitary gland (EPP) appearing in hyper signal T1 (thin white arrow) Height of the anterior pituitary gland: 2.5 mm.

**Figure 3:**
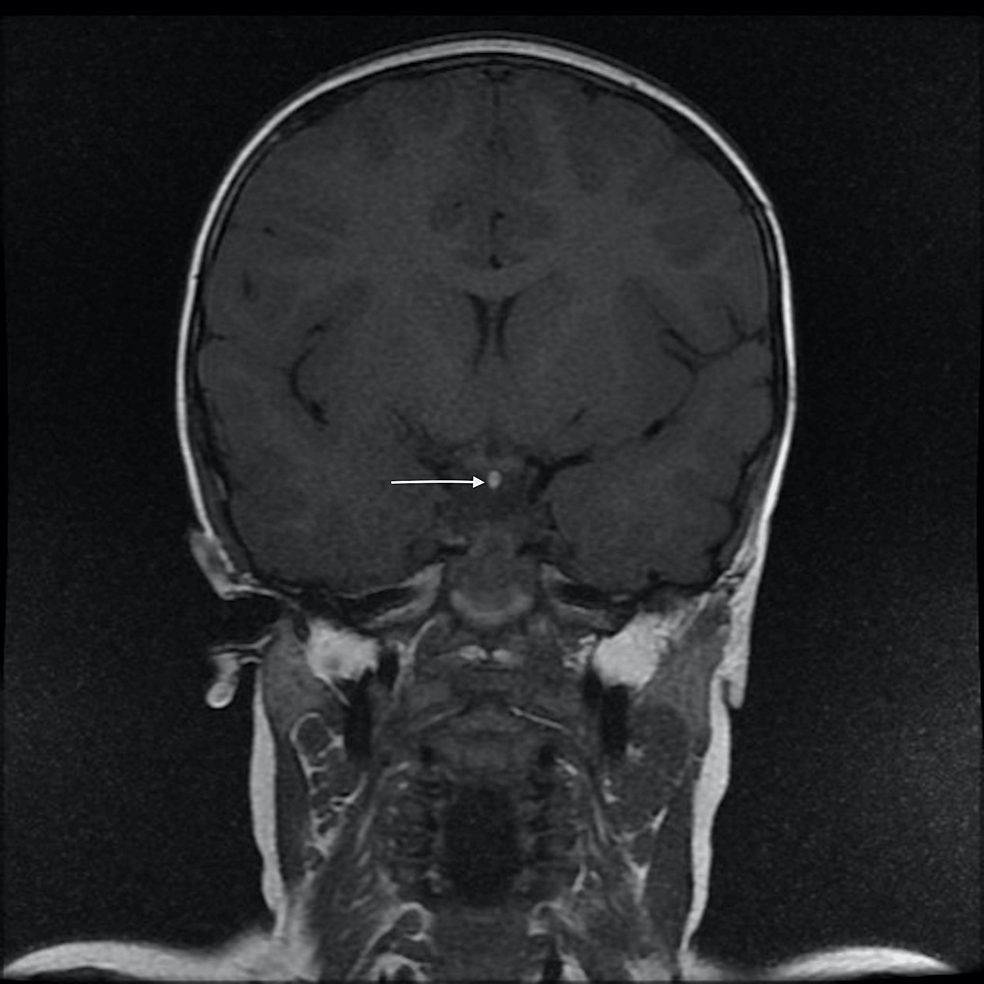
Coronal T1-weighted MRI section showing an ectopic posterior pituitary gland appearing in hypersignal T1 (thin white arrow)

**Figure 4:**
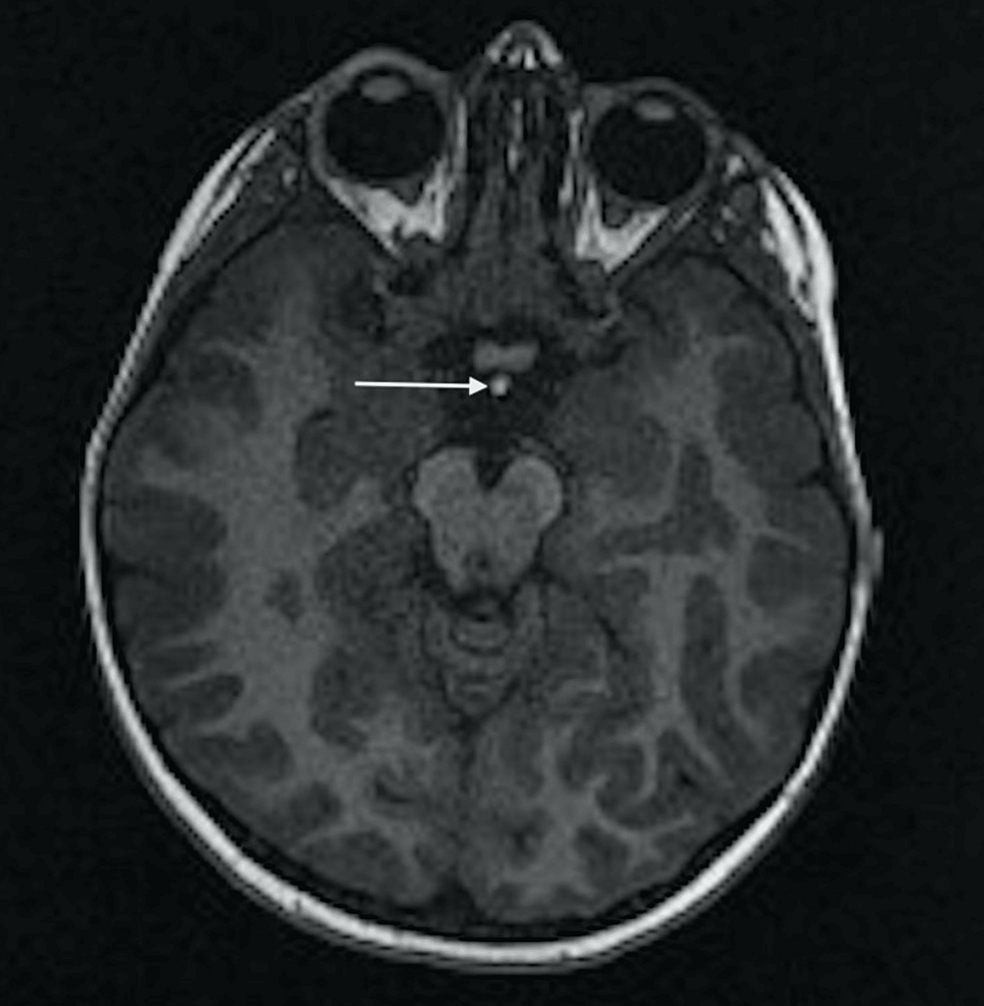
Axial T1-weighted MRI section showing an ectopic posterior pituitary gland found under the floor of the third ventricle, behind the optic chiasma

A mid-sagittal T1-weighted (T1W) MRI revealed an undersized sella turcica with a 2.5 mm tall hypoplastic adenohypophysis (thick white arrow) (Figure 2). The pituitary stalk was hypoplastic, unnoticeable, and limited to a small central remnant (dashed line arrow) (Figure 2). An ectopic posterior pituitary gland (EPP) appeared in hyper signal T1. It was found next to the hypothalamus median eminence, under the floor of the third ventricle (thin white arrow) (Figures 2-4).

These findings were consistent with PSIS. The PSIS diagnosis was retained on the following diagnostic criteria: hypoplastic anterior pituitary gland with a height < 3.5 mm, thin pituitary stalk (infundibulum), and ectopic neurohypophysis, appearing as T1W hyper-intense bright spot around the tuber cinereum. The patient started hormone replacement therapy with regular follow-up visits to a pediatric endocrinologist.

## Discussion

The PSIS, also known as pituitary stalk transection syndrome, is a very rare congenital entity [2], with an estimated incidence rate of 0.5/1,000,000 births [3]. It was first reported in 1987 by Fujisawa with the presence of an EPP being the cardinal feature together with an interrupted stalk [4]. There is a variable timing in the onset of clinical manifestations. PSIS is primarily encountered in infancy or childhood. Nevertheless, the diagnosis is often delayed [5]. The main symptoms of PSIS appear in the first decade of life. The most common clinical presentation is dwarfism. Growth hormone deficiency leads to growth retardation. Isolated growth hormone deficiency (IGHD) may evolve into multiple pituitary hormone deficiency (MPHD); this phenomenon can ultimately lead to pan-hypopituitarism. Nonetheless, the posterior pituitary function is usually maintained.

The PSIS diagnosis relies mainly on MRI, thanks to a characteristic triad [2,6]: hypoplastic or absent anterior pituitary gland (height < 3.5 mm), thin or absent pituitary stalk (infundibulum < 1 mm), and ectopic neurohypophysis, appearing as T1W hyper-intense bright spot around the tuber cinereum. This hypersignal is related to the presence of vasopressin vesicles and can be considered a marker of neurohypophyseal functional integrity. It contrasts with the anterior pituitary gland signal and the cerebral parenchyma. Most studies seem to show a good correlation between the presence of the hyper signal and the functional status of the post-pituitary gland [7]. Furthermore, the diagnosis can be evoked in front of an incomplete triad: an EPP or an interrupted pituitary stalk. Nevertheless, the diagnosis is certain when the triad is complete [2].

This affection may be associated with other cerebral congenital midline anomalies and other midline defects such as hypoplastic optic nerves and cleft lip palate [2]. The exact mechanism of PSIS remains unclear. Many etiopathological theories exist such as defective organogenesis during intra-uterine life or ischemic episode, forcing infundibular axon reorganization and ectopic posterior pituitary development [2]. Numerous reports have shown that there are many perinatal adverse events in PSIS patients, such as breech delivery, dystocia, and hypoxia [8,9]. Wang et al. studied 59 cases of children with PSIS, of which 54 cases had breech deliveries [10]. Fukuta et al. have supported the idea that breech presentation is a risk factor for pituitary stalk transection syndrome [11]. Recently, three holoprosencephaly (HPE)-related genes (SHH, TGIF, and SIX3) were found to be linked with PSIS. This finding implies that PSIS could be a mild clinical form of HPE [12,13].

Treatment of PSIS principally relies on lifelong hormone replacement therapy. Better prognosis and quality of life are linked to fast identification of hormonal deficiency with quick treatment initiation [14]. Wang et al. described in the literature the utility of increased first-year growth velocity as an effective predictor of final height in patients with PSIS [10].

## Conclusions

PSIS is a rare cause of pituitary dwarfism. The pathophysiology is not yet fully understood. However, this disease might be attributed to gene mutations and could be a mild clinical form of HPE. MRI remains the gold standard in the diagnosis process. It is characterized by a suggestive triad formed by the hypoplastic or absent anterior pituitary gland, thin or absent pituitary stalk, and ectopic neurohypophysis. When the triad is complete, the diagnosis becomes highly plausible. Treatment of PSIS principally relies on lifelong hormone replacement therapy. Early diagnosis enables quick treatment initiation and thus better prognosis and quality of life. Therefore, radiologists should be aware of this underdiagnosed entity with suggestive imaging features.
